# Palliative care for people with substance use disorders: a qualitative study of the experiences of rural primary care providers

**DOI:** 10.1186/s12904-025-01828-w

**Published:** 2025-07-23

**Authors:** Layale Tayba, Beatriz Cuesta-Briand, Kirsten Auret, Mathew Coleman

**Affiliations:** 1 WA Country Health Service, Albany, Australia; 2 The Rural Clinical School of WA, University of Western Australia, Albany, Australia; 3 Telethon Kids Institute, Nedlands, Australia

**Keywords:** Substance use disorder, Palliative care patients, Rural primary healthcare, GP experiences, Barriers to identification and management

## Abstract

**Background:**

In Australia, substance use disorders disproportionately affect people living in rural and remote areas. Patients with substance use disorders who receive palliative care have complex, often unmet, end-of-life needs. There is scarce evidence on the management of patients with substance use disorders in palliative care, and there is no consensus on the model of care to assist general practitioners manage their patients. This is particularly salient for general practitioners in rural areas, who provide most of the palliative care to their patients.

**Methods:**

This qualitative study explored the experiences of providing palliative care to patients with a substance use disorder among general practitioners in regional Western Australia. Data were collected through semi-structured interviews and subjected to thematic analysis.

**Results:**

A total of 12 interviews were conducted. Three main themes were identified: (1) a value-laden space; (2) substance-specific attitudes and practice; and (3) barriers to managing substance use disorders in palliative care. It was found that General practitioners’ personal values shape attitudes towards substance use, and impact on the management of substance use disorders for patients receiving palliative care. Attitudes and palliative care practice vary depending on the type of substance of concern. Perceived barriers to recognition and management of substance use in palliative care included patient, health professional, health system and societal factors.

**Conclusions:**

Early recognition and intervention for people presenting with substance use disorders in palliative care settings may improve patient quality of living and make managing life-limiting illness safer and more effective. In rural settings in Australia, care is often provided by general practitioners yet despite their whole of person approach to medicine and capacity to manage complexity and multimorbidity, challenges persist in optimising the care of substance use disorders in palliative care. This study provides insight into areas for further research, the potential for the development of clinical practice standards and guidelines in this complex area of palliative care.

**Supplementary Information:**

The online version contains supplementary material available at 10.1186/s12904-025-01828-w.

## Background

Substance use disorders (SUD) is a collective term that describes the ongoing use of drugs or alcohol despite consequential impairment of an individual’s functional, social and emotional well-being. Much like other chronic illnesses, SUD can persist throughout the lifespan [1, 2] and result in considerable health and social burden on the individual and society more broadly [3–5]. SUD often co-occur with psychiatric disorders including mood, anxiety, psychotic and personality disorders [4, 5], and studies have identified SUD with comorbid mental disorders as the leading cause of disability globally [6–8].

For people living in regional and remote areas SUD burden increases with remoteness leading to poorer health outcomes, higher levels of disease and shorter lives [9–11]. People in rural Australian communities are twice as likely to smoke tobacco, drink alcohol at harmful levels and consume illicit substances at higher rates than people in metropolitan areas [9, 12, 13]. Access to health services including mental health professionals and alcohol and drug services decreases with increasing remoteness, contributing to the poorer health outcomes for rural people [9–11] including premature death [14–20].

Patients with SUD in Palliative Care (PC) have complex, often unmet, end-of-life needs [21]. PC is a field aimed at improving Quality of Life (QOL) and reducing suffering of patients and their families facing life-threatening illness [22]. It is well established that people receiving PC show improvements in QOL, reduced symptom burden and higher patient/carer satisfaction [23–25]. PC is commonly accessed by older adults, a population not immune to SUD, including tobacco, alcohol and illicit drug use [9, 12, 26]. SUD within PC has multiple aetiologies including SUD developing during PC (e.g. opioid use) [27, 28], terminal illness directly related to SUD (e.g. alcohol-related liver disease) [29–31], or comorbid SUD unrelated to the terminal illness.

General Practitioners (GPs) are the backbone of care in rural settings, including PC, and often provide care that elsewhere may be referred to a specialist [32–42]. Benefits of GP-led care include pre-established relationships with patients and their families promoting continuity of care and increased understanding of local culture and issues affecting their communities [33, 36]. There continues to be barriers to patients receiving care for SUD from GPs including stigma and perceived lack of education and training [43–46]. Emerging research highlights the overlap of skills required for delivery of PC and SUD treatment, suggesting rural GPs could be well positioned to address the complexity of co-existing SUD in their PC patients [47, 48].

Despite crossover, literature on managing SUD in PC remains limited [49, 50] with emphasis on symptom control, medications with potential for abuse or diversion and SUD prevalence [51–66]. Data is emerging on the biopsychosocial needs of alcohol dependency in PC [26] and a limited body of research on the homeless as a specific high-risk population [67–69]. Barriers to accessing quality PC include fears of abuse and diversion of medications, stigma and ‘drug seeker’ labelling leading to suboptimal assessment and management [21, 57, 70–76], patient distrust and clinicians’ lacking training [77–85]. Although qualitative research including patients, families and care providers of people with SUD in PC is emerging [78–84], and a recent systematic review [85], this data is restricted to large metropolitan areas outside Australia limiting generalisability across treatment settings [70]. To date, no consensus model of care exists to assist GPs in providing treatment to people with SUD in PC, let alone within rural contexts.

The study reported here explored the attitudes, challenges and approaches of rural GPs in managing patients with co-existing SUD and PC needs, providing a deeper understanding of the management of these patients within a primary care setting which will hopefully contribute towards the development of a cohesive model of care.

## Methods

This was a qualitative study informed by phenomenological theory, insofar as it was interested in understanding the experiences of GPs delivering PC in a rural area of Australia. The study adhered to the COREQ (consolidated criteria for reporting qualitative research) checklist [86]. Ethics clearance was granted by the Human Research Ethics Committee of University of Western Australia (*2021/ET000203*). Informed consent was obtained and recorded from all study participants.

### Sampling and recruitment

The sample was recruited among GPs (including GP Registrars) who had provided PC in the community to at least one patient with co-existing SUD (either as defined by the DSM- [87] or informally assessed) over the last 12 months, and/or were credentialed to admit to the local hospice. A purposive sampling technique was adopted to recruit eligible participants. GPs were recruited through the distribution of project flyer and information sheet via practice managers and professional networks. Furthermore, a snowball sampling technique was adopted. Recruitment ceased once data saturation was achieved. Participation in the interview did not include reimbursement of any form.

### Data collection

Data was collected by author LT through semi-structured interviews conducted between August and December 2021. The interview schedule was specifically developed for this study and consisted of open questions and prompts designed to explore participants’ experiences and attitudes towards providing PC to people with SUD (see supplementary material). The schedule was piloted to refine the questions. Participants were invited to discuss their conceptualisation, challenges and approach to managing patients with SUD receiving PC. The interview schedule is available from LT, however the dataset generated and analysed are not publicly available due ethics and privacy issues but are available from the corresponding author on reasonable request. The interviews were conducted by telephone or face-to-face. Consent was provided in writing prior to the interview. Interviews were audio recorded and transcribed verbatim.

### Data analysis

De-identified interview transcripts were subjected to thematic analysis [88, 89]. The research team included a trainee psychiatrist working rurally with experience in both PC and addition psychiatry, an academic consultant psychiatrist who is an addiction specialist, a palliative medicine academic physician, and a research fellow with expertise in qualitative research methodology. All researchers began by independently reading and re-reading transcripts to immerse themselves in the data and identify initial patterns. Team members examined their own beliefs, judgements and practices during this process and shared these reflections at meetings to consider if, and how these may influence the research. A relevantly skilled interdisciplinary research team in combination with review of the existing literature was intended to enrich the analysis and promote interpretive rigor [90–92]. Through this iterative process initial codes were derived by manually examining and tagging the data by highlighting key words, sentences and/or paragraphs. Codes were grouped into broader themes and subthemes, which were repeatedly refined by the authors in a reflexive approach until consensus was reached. Three main themes were clearly defined and named to ensure they accurately reflect the underlying meaning. After twelve interview transcripts were analysed, no new codes emerged, reaching data saturation [93]. 

## Results

A total of 12 GPs were interviewed as seen in Table 1. The interviews had an average duration of 39 min.

Thematic analysis of the interview transcripts yielded three main themes: a value-laden space; substance-specific attitudes and practices; and the challenges of managing SUD in PC. These themes are discussed below and are accompanied by anonymized, illustrative quotations.

**Table 1:**
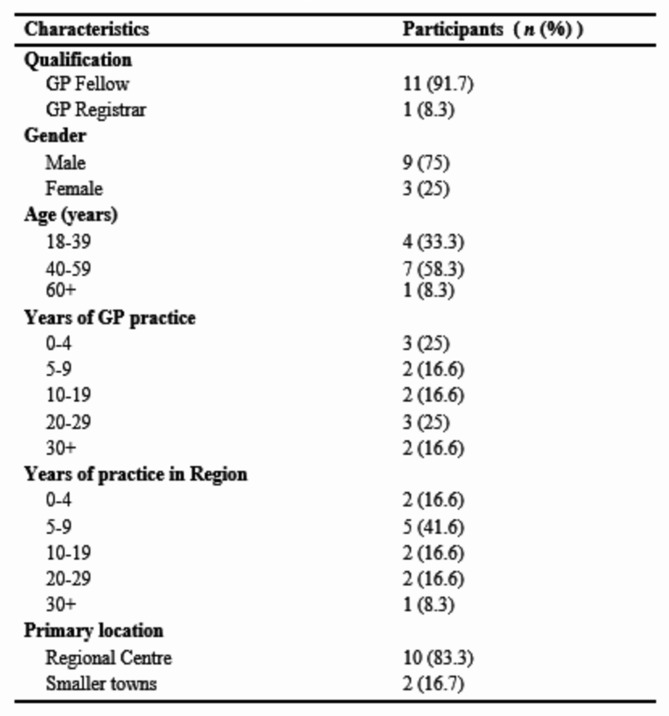
Characteristics of interview paticipants

### A value-laden space

Implicit and explicit references to personal and societal values permeated participants’ accounts. Interview data illuminated ways in which values shaped attitudes towards and perceptions of patients’ psychosocial circumstances and impacted on the clinical management of SUD in PC.

#### Tension between non-judgmental practice and implicit/explicit judgments

GPs’ accounts of their attitudes towards substance use, SUD and its management in PC, revealed a tension between the goal of delivering empathetic and non-judgement care and internalised value-laden judgements and assumptions. Most GPs spoke about striving to be empathetic and non-judgemental, characteristics that were seen as cornerstones of primary care practice and regarded as being of even greater relevance to PC. GPs described PC as a ‘special’ field of practice, characterised by a compassionate, person-centred, holistic and multidimensional approach including physical, but also social, spiritual and psychological components and interventions. Thus, PC was perceived as being ‘different’ from traditional ‘hospital medicine’:

‘That’s why palliative care’s so different. Everything’s open for discussion and what do you want us to treat and how do you want us to manage this, which context do you want to do it in? It’s wonderful to have such patient-centred management, and also family. It’s all inclusive. But it’s such a paradigm shift from the way we practise, hospital medicine at least.’ (I04, Male, 20–29 Yrs Practice).

GPs’ common goal of providing holistic, non-judgemental, empathetic care appeared to be in contradiction with implicit value judgements passed on certain patients and behaviours, which were evident in the accounts of some GPs, for example in the language used to refer to patients with a SUD (‘meth heads’, ‘pot heads’, ‘boozers’) or the characterisation of some substance users as ‘irritable’, ‘chaotic’ or ‘difficult’. One GP, who admitted to having limited experience managing substance use in PC commented:

‘… some palliative care patients are relatively easy and very pleasant and nice to look after. Patients with substance abuse are generally not nice patients. They don’t say thank you, they’re not always nice to the staff, they’re more inclined to complain. That’s my limited experience.’ (I11, Male, 30 + Yrs Practice).

Some GPs referred to societal attitudes towards certain substances (‘illegal drugs’ such as methamphetamine were often cited) and towards certain behaviours, and how these shaped perceived attitudes observed in others. Thus, some GPs tended to distance themselves from admitting having prejudices and attributed judgemental attitudes to other colleagues, including doctors and nurses:

‘So, of course, some people, especially in the area of illegal recreational drugs but even alcohol, I suppose, or smoking – some people can have a very judgemental attitude and there’s just very unhelpful black and white attitudes, I suppose, about patients and how they came to be in the situation that they’re in. And so, I suppose – and again, just speaking from my own experience, I think there’d be a small minority of doctors that might struggle to offer compassion and holistic care because they’re coming from that sort of more moral space perspective.’ (I12, Male, 30 + Yrs Practice).

Similarly, another GP reflected on the attitudes of others:

‘So, I find that people seem to be just a bit less sympathetic sometimes if there’s a perception that that person’s substance use has contributed to their condition. So, like lung cancer or COPD in someone who’s a heavy smoker, for example, there does seem to be a bit less of that sense of – I don’t know. People seem to think it’s much more distressing if you have lung cancer and you’ve never smoked, as opposed to if you have lung cancer and you’ve smoked. They seem to – yeah. There just seems that perception that – not so much that people deserve it, but that it’s a consequence that they chose to take the risk on.’ (I05, Female, 10–19 Yrs Practice).

Although some accounts suggested a certain level of deflection, attributing judgemental attitudes to society at large or to other colleagues, other GPs acknowledged that everybody had their own prejudices, and some admitted to occasionally having to reign in their ‘judgemental self’ when managing patients with SUD. One GP explained:

‘…I would have to be putting on my very GP, non-judgmental, caring, protective arm, to look after them in a good way. Rather than letting my emotional self and judgemental self say they shouldn’t be using up our services. So, I’d have to try hard. But I would do it. (I01, Female, 20–29 Yrs Practice)

Reflecting on whether the type of substance influenced their attitude, one GP openly admitted:

‘Well, I think I treat people differently. I mean, I’m a little ashamed to say that, but I think I just treat people differently.’ (I11, Male, 30 + Yrs Practice).

#### Perceptions of patient characteristics

Generalisations and assumptions about the profile of the PC patient cohort and the ‘typical’ presentation of substance users were common among GPs and influenced clinical decision-making, especially screening. GPs commonly described their PC patient cohort as older patients with chronic conditions, with the notable exception of one participant who reported only seeing Indigenous patients among whom the PC cohort had a younger profile. Some accounts demonstrated that, based on these characteristics, there was an assumption that their patients were more ‘less likely’ to fit the risk profile for substances other than alcohol and tobacco:

‘I suppose in my cohort, it’s predominantly alcohol, and smoking, I think. We have a very elderly patient group in our community, and of course, in the palliative care setting, that most of our patients are reasonably aged. So, we’re not sort of seeing a lot of the typical socially ‘ice’, or a lot of the more recreational drugs and whatnot.’ (I08, Male, 20–29 Yrs Practice).

Similarly, another commented:

‘Most of my palliative people are elderly and I really doubt there’s a lot of information to be gained if someone’s dying of bowel cancer or whatever and they’re 80. The chance that they’re dope smokers at all let alone of any sort of frequency that’s going to impact significantly is very low.’ (I12, Male, 30 + Years Practice).

The quotes above reflect the broadly held view that PC patients were a low-risk cohort with regards to using substances other than alcohol and nicotine. Assumptions made about behaviours associated with patient characteristics (mainly age) influenced attitudes towards addressing substance use in PC and screening for substance use in the first place.

Value-laden attitudes and perceptions were also evident in some GPs’ references to the ‘typical’ presentation of a ‘substance user’ – this phrase tended to be used to refer to users of substances other than alcohol and nicotine. Whilst comments demonstrating strong negative attitudes were the exception (‘meth heads look like meth heads’), GPs acknowledged that attitudes and perceptions had the potential to influence clinical practice, especially around recognition and screening:

‘I think sometimes people have a perception of what a person with a substance use looks like or sounds like or acts like. So, I think that would be a barrier, because sometimes people just wouldn’t think to ask the question. Or would just assume things that aren’t necessarily true.’ (I05, Female, 10–19 Yrs Practice).

Overall, GPs who reported being more experienced in managing substance use beyond alcohol and nicotine in a PC setting and in primary care more broadly tended to have more nuanced views and a greater understanding of the complexity of substance use in the community.

### Substance-specific attitudes and practices

GP attitudes towards, and practices around substance use were strongly influenced by the type of substance and the underlying social attitudes attached to each type of substance. GPs’ accounts suggested that there is a continuum of perceived social acceptability, with tobacco and alcohol at one end and methamphetamines at the other, that is often, but not always, inversely correlated with perceived patient care complexity.

#### Characterisation of substance use in palliative care

GP were asked to reflect on how they characterised substance use issues in the PC setting. Whilst a minority admitted that they did not have that sort of mental ‘structure’ (I02, I03), most referred to perceived categories of substances that influenced their attitudes and practices. In this context of substance categorisation, GPs spoke of ‘socially acceptable or not’, ‘legal or illegal’, ‘harmful or not’, ‘helpful/resourceful or unhelpful/unresourceful’ or ‘problematic or not’. Alcohol and tobacco were widely regarded as ‘common’ and ‘socially accepted’ and, typically, their use in the context of PC was seen as less ‘problematic’. One GP reflected:

‘Often our patients’ do self-medicate a little bit with alcohol, which I don’t really see a problem with. A lot of people drink – and I’m not a GP to really labour that point, especially in palliative care patients.’ (I02, Female, 0–4 Yrs Practice).

At the opposite end of the spectrum, illegal substances, particularly methamphetamine and, to a lesser extent, heroin, were broadly conceptualised as problematic, irrespective of markers of harm:

‘So obviously if somebody is on an illegal drug, so say they were a meth user, I’m not going to be able to support them… in the way that we could sign a form for someone to go out and smoke a cigarette. Or we can keep alcohol in the medicine room at the nurses’ station.’ (I05, Female,10–19 Yrs Practice).

In the context of PC, and despite its status as a prohibited drug in Western Australia, recreational cannabis use was often categorised as ‘helpful’ or ‘resourceful’ due to its perceived properties:

‘A lot of pal[liative] care patients just smoke cones[cannabis] and they have only ever done that since they have bone pain or visceral pain, as an alternative, and I think that’s okay.’ (I04, Male, 20–29 Yrs Practice).

Similarly, another GP explained:

‘Resourceful use, for me, would be someone who has been a long-term cannabis user and now has metastatic cancer and finds relief from smoking cannabis. I think that’s fine. We went to [a patient’s] house […]. She had metastatic uterine [cancer] and she could only roll over and she was smoking buckets. She could only roll over and she’d have her morphine, a fentanyl patch and everything, but smoking cannabis for her was what did it and she was calm and she was fine. I think that’s resourceful use. (I10, Male, 5–9 Yrs Practice)

Although substance use in PC was largely characterised based on the type of substance involved, some GPs noted that the main aspect influencing their practice, irrespective of the type of substance, was whether the use was a perceived problem for the patient or from the doctor/staff point of view. One GP explained:

‘I categorise [substance use disorders] as a perceived problem for the patient or not a perceived problem for the patient, which isn’t a traditional way of categorising. But – as in does the patient feel that there’s an issue that we need to manage? Or is the patient going, “oh, this isn’t a problem”? And then from the doctor or nursing staff point of view, do we feel that there’s a problem? For example, the patient that was drinking a bit too much in his room, we were quite concerned about frequency of falls, and combining with other pain medication and things.’ ((I05, Female, 10–19 Yrs Practice).

#### Impact on clinical management

Given the complexity of PC management and its patient-centered approach, generally there was little impetus to address substance use unless this impacted on clinical management; this fitted in an end-of-life context where priorities regarding lifestyle and behaviour shifted from the ‘normal conversation’. GPs’ accounts of the impact of substance use on PC management was strongly influenced by the type of substance use and its perceived potential harm to self or others, and modulated by the PC setting. The following quote of one GP reflecting on the clinical decision-making process around whether or not to address substance use in PC illustrates these points:

‘… depending on their timeframe and their goals of care, if it’s something that they really want to do, or if they’ve got a terminal condition, do they want to address it now? Or do they want to continue how they are? Or if they’re continuing, will that cause significant to harm to them? Then on top of that, then you’ve got to look at the ability for them to be admitted either into the hospice or a hospital; what that particular substance is, whether that will be acceptable to the institution, and what we would do if they had to be admitted.’ (I01, Female, 20–29 Yrs Practice).

Harm to self was broadly articulated as interfering with symptom control measures. In this context, tobacco smoking was largely seen as ‘ok’ and not worth addressing unless it interfered with oxygen therapy, in which case nicotine replacement therapy was required for the patient to be placed on oxygen safely. This relatively laissez-faire attitude towards smoking was challenged when patients received PC in the hospice setting given the current strict ban on smoking on all healthcare facilities, including hospices. One GP explained that, until recently, hospice patients were allowed to smoke in the garden or at the front of the building, adding:

‘But the rules have changed, so they can’t. And the nearest place is about 200 metres away, and our smoking population get too short of breath to get that far’. (I03, Female, 10–19 Yrs Practice)

GPs spoke about patients discharging themselves or hesitating to be admitted because of this policy change, and some comments suggested an internal conflict between a rational understanding of the need for the ban and a desire to allow their patients to smoke. This was aggravated by a perceived increasing cohort of smokers with chronic obstructive pulmonary disease requiring PC. The management of alcohol use broadly elicited a similar non-interventionist response, as this quote illustrates:

‘So if someone drinks four shots of whiskey every night and is dying of heart failure, I’m not stressed about them continuing to drink, provided they see that as something that they are going to keep doing. (I04, Male, 20–29 Yrs Practice)

In contrast with cigarette smoking, GPs reported being able to manage alcohol use in the hospice setting relatively easily, as alcohol could be included in the patient’s medication chart. With regards to the use of opioids, the major impact on clinical management reported was derived from patients not being opioid naïve and potentially requiring further up-titration for pain control compared with opioid naïve patients. This was irrespective of how the person had developed the opioid tolerance or dependency, and also included those whom one GP described as ‘clockwatching’:

‘Usually a frail, elderly person or a 60- to 70-year-old person with a chronic non-cancer disease who becomes quite dependent on a substance to try and medicate themselves. I don’t know if you’d call that substance abuse but - or clockwatching. Even in a nursing home, it happens, a patient who is really quite distressed and really needs their opioid and ringing the bell on the hour, every hour, to get their medicine. That’s very common. That is something that we do see quite a lot of.’ (I10, Male, 5–10 Yrs Practice).

Among the sample of GPs interviewed, potential risk of harm to others, including family, staff and society at large, was perceived as a trigger for intervention and/or a disruptor to clinical management, and there was strong consensus about opioids and methamphetamines potentially being the most ‘harmful’ and bringing the greatest amount of ‘chaos’ and ‘disruption’ to patients and people around them. One GP reflected:

‘I guess from my experience, just the amount of disruption to the person and the people around them, the alcohol and the cigarettes, and to an extent, pot, don’t tend to have as big an impact, that I’ve seen, on patients and their family. Opioid and methamphetamine seems to crash the pack, so to speak.’ (I06, Male, 5–9 Yrs Practice).

The quote above broadly reflects a commonly held view among the GPs interviews regarding the perceived potential harm to others associated with different types of substances, however, some highlighted the behavioural and social issues surrounding problematic alcohol use, which could put staff at risk both in the hospice and in the community, as this GP explains:

‘It might not be safe for a nurse to go out to that house. I mean, that’s certainly been the case with people who have alcohol issues, that we’ve had basically issues that the house has been unsafe to go to. It’s there’s dog shit everywhere, and that sort of scenario we had last year with a guy who was drinking, who was being palliated 20 Ks from town. And the nurses eventually had to say, “No, we can’t do this.’ (I08, Male, 20–29 Yrs Practice).

### Barriers to managing substance use disorders in palliative care

The interview data suggests that managing substance use in PC is broadly experienced as a layer of complexity adding to the already complex, value-laden palliative care space. GPs’ accounts show that SUD may remain unrecognised and/or unaddressed; when SUD is explicitly acknowledged, their management may be disruptive, altering care pathways.

#### Recognition and screening of substance use disorders

GPs were explicitly invited to identify potential barriers to recognising substance use disorders in the palliative care context. As seen in Table 2, reported barriers, both perceived and experienced, included patient, health professional, health system and sociocultural factors.

**Table 2:** GP-Reported key barriers and challenges impacting on the identification of SUD in PC

Barriers/Challenges		Supporting Statements
**Patient Factors**	• Non-disclosure (or incomplete disclosure) due to perceived impact on care • Feelings of discomfort or embarrassment due to perceived stigma	*‘Even though we think we’re asking the good*,* non-judgmental questions*,* we have to live in the real world. People actually don’t tell us the truth. People may think that they may not get good care if they admit that they’re using substances.’ (I01*,* Female*,* 20–29 Yrs Practice)*
**Health Professional Factors**	• GP lack of awareness or oversight. • Perceived lack of expertise • Fear of embarrassing patients • Low prioritisation due to breadth-focus (coverage of all competing distresses in PC)	*‘I think there are some doctors*,* I hate to say*,* that are probably in denial of the extent of the problem or feeling that it’s not their problem or that they are not experienced in that area or will not be able to handle the situation well*,* or there will be little or no support. Sometimes it’s easier to bury your head in the sand.’ (I11*,* Male*,* 30 + Yrs Practice )* *‘I mean some people could potentially be offended by – if you ask someone*,* “Do you use any recreational or illegal or non-prescribed drugs?” some people will feel that there’s an implicit judgement in that question. Although I suspect that we as clinicians feel the risk of that higher than it is amongst the patient population.[…]there’s such a blooming multitude of things you’ve got to cover and do anyway. So I guess you can’t see the trees for the forest sometimes. Realistically*,* you’ve got to prioritise what you think are the important things to raise.’ (I12*,* Male*,* 30 + Yrs Practice)*
**Sociocultural Factors**	• Stigma around SUD • Societal perceptions around acceptability of use (or not) for certain substances.	*‘They’re quite a vulnerable population*,* they’re nearing the end of life*,* it’s quite a sad time for a lot of them – they don’t want to be labelled as a drug addict at the end of their life.’ (I02*,* Female*,* 0–4 Yrs Practice)* *‘There’re things that we find more acceptable. Alcohol probably causes more harm*,* but it’s totally acceptable in our community.’ (I01*,* Female*,* 20–29 Yrs Practice)*

As seen in Table 2, GPs identified a broad range of potential barriers and challenges to the identification of SUD in PC. In addition, and although not cited by GPs as a barrier per se, GPs’ description of their practices around substance use management revealed that an issue impeding the effective identification of substance use in PC was the lack of a systematic approach to screening (not ‘routinely asking the question’), which could be categorised as a health system factor. When invited to reflect on potential barriers to the identification of SUD, one GP with 5-years’ experience working in PC full time said: ‘I don’t think there’s any barriers. It’s just that we don’t think about it.’ Later, this GP commented:

‘… you’ve brought up a really good point because I’ve never asked anyone about [substance use]; never asked them how much they smoke; what they smoke; if they’re taking any other substances. Never.’ (I10, Male, 5–9 Yrs Practice).

Similarly, another GP reflected on the ‘jokey’ approach to broaching the subject of substance use:

‘I think the patient not bringing it up, and us probably not asking. Or having a bit of a more jokey, colloquial, “okay, they like a joke,” or whatever, but not really asking specifically how much they drink and how often, and all that kind of thing.’ (I05, Female, 10–19 Yrs Practice).

Many GPs reported assessing potential SUD in new patients as part of the social history, however, the majority reported not using specific screening tools. Furthermore, these initial assessments did not necessarily reflect the true extent of the substance issue and its change over time, so that it often only became apparent in PC at crisis points mostly triggered by symptom control issues.

Interestingly, personal attitudes towards substance use, and assumptions made about patients and about the ‘typical’ presentation of a patient with a SUD were not mentioned by GPs as potential barriers to screening.

#### On-going management of substance use disorders

Interview data revealed that, even when SUD are identified, there are barriers to their ongoing management in the PC space. These barriers are summarised in Table 3 and, similarly to GP-identified barriers to recognition and screening, they include patient, health professional, health system and societal factors.

**Table 3:** GP-Reported barriers and challenges to on-going management of SUD in PC

Barriers/Challenges		Supportive Statements
**Patient Factors**	• Patient wishes	*‘…if it was to get themselves cleaned up*,* well*,* then great. Let’s help them do that. If it’s not*,* then we’ll focus on the other areas that they feel will make a difference to them.’ (I12*,* Male*,* 30 + Yrs Practice)*
**Health Professional Factors**	• Ambivalent attitudes towards the value of addressing substance use in PC • Perceived lack of confidence & expertise	*‘I guess when a patient is at the end of life*,* and may have days to weeks to live*,* you don’t really care about the long-term negative effects. Socially*,* physically*,* on their stigma*,* on their family -because they are going to die soon*,* and comfort is probably the most important thing.’ (I02*,* Female*,* 0–4 Yrs Practice)* *‘So personally*,* I’ve done a little bit of extra training in substance abuse and alcohol use disorders. And so I guess it’s an area I think a little bit more about sometimes*,* and have a little bit more experience in managing. Whereas I think if you don’t have the experience*,* you tend to shy away from treating it a bit more.’ (I05*,* Female*,* 10–19 Yrs Practice)*
**Health System Factors**	• Perceived scarcity of support & rehabilitation services	*‘I think it’s a specialist area that needs high-intensity care*,* and…I don’t think we have really good*,* easy-to-access facilities that don’t have a really long waitlist or that are culturally safe… So I’m finding it’s a bit hard without the backup of really good services to do a proper job as a GP. (I03*,* Female*,* 10–19 Yrs Practice)*
**Sociocultural Factors**	• Stigma	*‘I’ve just had some experiences at times when we’ve had people in for detox or other things*,* that sometimes people who haven’t worked in drug and alcohol spaces before*,* or maybe don’t have much personal or family experience*,* can be quite judgmental.’ (I05*,* Female*,*10–19 Yrs Practice)*

## Discussion

The study reported here explored the attitudes, challenges and approaches of rural GPs in managing patients with co-existing SUD and PC needs, providing a deeper understanding of the management of these patients within a primary care setting which will hopefully contribute towards the development of a cohesive model of care. Our findings align with other studies suggesting that the recognition, management and approach of medical professionals, in this case rural GPs, is incomplete in many PC circumstances [79–85]. Further, GPs conceptualised SUD as adding complexity and ‘problematic’ in PC.

Healthcare professionals are crucial in the identification of people experiencing SUD and in the access of health care and treatment for people experiencing SUD. What was indicative in our study findings was the strong pre-existing value-laden prejudices that can impact GP responses and care in PC with people experiencing SUD. Potentially modified by patient behaviour (or anticipated behaviour through stereotyping), the interaction between a patient with a SUD and GP is described as predictable, negative and potentially manipulative rather than a complex interplay between intoxication, withdrawal, addiction, illness behaviour, negative attitudes, stereotyping or discrimination [94]. However this effect was not evenly distributed across a SUD patient cohort, with the substance in question playing an important mediating factor for GPs. Simplified good versus bad heuristics were utilised to manage the complexity of substance, patient, health provider and systems issues. Unsurprisingly these findings are not unique to PC in healthcare systems for people experiencing SUD.

Recognition and screening for SUD in PC in our study was minimal, considered ineffectual or even irrelevant, despite ample evidence for the benefits of early interventions, particularly on QOL in PC [95, 96]. This finding is in line with other studies demonstrating the utility of developing system wide clinical guidance. The development and refinement of PC-wide SUD screening tools that account for pre-existing, developing or iatrogenic SUD will be key areas for advances in this under-recognised and complex clinical area. Adapting existing SUD related screening tools such as the AUDIT, DUDIT or Opioid Risk Tool into system wide standards of care may be a simple and effective avenue to improve the attention of care for SUD [97].

Similarly, further research into developing standardised approaches to managing SUD in PC would invaluably contribute to the dearth of clinical practice guidelines for PC clinicians and patients alike. One of the challenges in PC is framing practice guidelines in the context of often short and evolving poor prognoses amongst other mounting treatment burdens and priorities [98]. Episodes of best practice care may be brief and complex due to the interplay of multimorbidity, illness effects and treatment demands that occur during a dynamic period of deterioration towards death [99]. Yet in spite of the addition of SUD to the issue of existent complexity in PC, clinical practice guidelines can acknowledge and enhance clinician and patient decision making by translating complex scientific research findings into recommendations for clinical practice that are relevant to the individual patient encounter, instead of implementing a one size fits all approach to patient care [100]. 

Adding patient and carer lived experience, including the potential benefits of peer worker contribution in PC settings would be important first steps in overcoming the negative value-laden space that is experienced by people with SUD in PC. PC is not without an empathic and engaged workforce, and our study participants demonstrated this by their longevity and commitment to the field. Overcoming suboptimal care associated with stereotyping and discrimination of people with SUD is not confined to PC, but more generally across all healthcare settings. Aligning existing standards of PC provided by GPs that is collaborative, holistic, patient-led and inclusive of carers and family remains the hallmark of excellence in PC.

Our study, however, is not without limitations, particularly with respect to generalisability in view of its rural setting, its GP focus and the nature of the small sample size. Despite this, participants shared their experiences openly with prolonged engagement improving credibility, consequently providing rich data despite low numbers. The researcher’s predispositions require acknowledgment, undoubtedly bringing our own experiences into data analysis, increasing risk of investigator bias. Contrariwise involving topic experts in the interpretive process is considered a strength of hermeneutic phenomenology. Interviews evaluated experiences from a GP perspective to the exclusion of other healthcare workers, patients, families or carers. Expansion to include additional cohorts would provide greater diversity to our findings. The above ensured project feasibility, however caution regarding transferability of results is required, particularly to urban settings with greater access to specialists and tertiary care.

## Conclusion

Early recognition and intervention for people presenting with SUD in PC settings may improve patient QOL and make managing life-limiting illness safer and more effective. In rural settings in Australia, care is often provided by GPs yet despite their whole of person approach to medicine and capacity to manage complexity and multimorbidity, challenges persist in optimising the care of SUD in PC. This study provides insight into areas for further research, the potential for the development of clinical practice standards and guidelines in this complex area of PC.

## Electronic supplementary material

Below is the link to the electronic supplementary material.

Supplementary Material 1

## Data Availability

The interview schedule is available from the LT (corresponding author) however the dataset generated and analysed during the current study are not publicly available due ethics and privacy issues but are available from the corresponding author on reasonable request and amended ethics approval.

## Abbreviations

COREQ: Consolidated Criteria for Reporting Qualitative research
GP: General Practitioner
PC: Palliative Care
QOL: Quality of Life
SUD: Substance Use Disorder/s
