# Supplementary material for: Palliative care for people with substance use disorders: a qualitative study of the experiences of rural primary care providers
Source: BMC Palliat Care. 2025 Jul 23;24:210. doi: 10.1186/s12904-025-01828-w (PMC12285175; doi:10.1186/s12904-025-01828-w)
Supplement: Supplementary file 1 — Supplementary Material 1 [file 12904_2025_1828_MOESM1_ESM.docx]

**Appendix: Semi Structures Interview Questions. Palliative care for people with substance use disorders: A qualitative study of the experiences of rural primary care providers**

1. **Let’s me start by asking you to share your professional experiences with providing palliative care?**
   1. Prompting questions if needed 🡪 length of practice, level of experience, perceived confidence with service provision, what services they provided and in what settings.
2. **Please tell me of your professional experiences in providing substance use disorder treatment in primary care?**
   1. See above prompting questions if needed
3. **Let’s now think about the cooccurrence of the two topics – that being people who experience substance use problems in the context of requiring palliative care. Before I ask you any specific questions, I’d like you to broadly reflect on this cohort.**
4. **Now, can you share a brief summary of your professional experience managing patients who have co-occurring palliative care needs and substance use problems?**

1. **Reflecting on your own practice, how do you think about or characterise substance use problems in palliative care? Discuss how this impacts your experiences or attitudes?**

1. **Reflecting on your own practice, discuss how and when people with substance use problems in palliative care are being identified.**
   1. Answer may to lead to further exploration 🡪 why might this be the case? Discuss the barriers to identification/screening?
2. **Can you share your management approach(es) towards providing treatment to patients experiencing substance use problems in palliative care?**
   1. Prompting questions if needed 🡪 What might be some of the patient’s needs? how do you approach/manage patient’s care needs? Examples? describe your priorities when it comes to providing treatment? Is addressing the substance use important?
3. **Are you able to share your professional experiences with moving towards the end stages of life and potential transition from home-based palliative care to hospice care for people with substance use problems?**
   1. Note 🡪 if participant is not familiar with hospice, focus remains on end-of-life care delivered in the community
   2. Prompting questions if needed 🡪 Does your management approach or priorities shift with respect to symptoms control, addressing the substance use etc?
4. **Reflecting on your practice of providing palliative care to people who experience substance use problems, please discuss any particularly positive or challenging experiences?**
5. **Finally, can you discuss how does your level of knowledge or competency with these areas of practice influence your attitudes and practice**?
   1. Prompting questions if needed 🡪 have you received adequate training on this topic, how would they describe their level of competence, does this impact on their practice?
6. **There are no further interview questions but at this point I would like to ask if there are any further comments you would like to make?**
